# Transvenous Catheter-Based Thrombolysis With Continuous Tissue Plasminogen Activator Infusion for Refractory Thrombosis in a Patient With Behcet’s Disease

**DOI:** 10.7759/cureus.10049

**Published:** 2020-08-26

**Authors:** Saeed Arif, Shaheer Arif, Jahanzeb Liaqat, Atiq-ur-Rehman Slehria, Abdur Rahim Palwa

**Affiliations:** 1 Neurology, Pak Emirates Military Hospital, Rawalpindi, PAK; 2 Radiology, Armed Forces Institute of Radiology and Imaging, Rawalpindi, PAK

**Keywords:** behcet’s disease, cerebral venous sinus thrombosis, arteriovenous thrombosis, inferior vena cava filter, catheter-directed thrombolysis

## Abstract

Behcet’s disease (BD) classically presents with recurrent oral ulcers, genital ulceration, uveitis and skin manifestations. Middle-aged people are usually affected with the male gender being associated with severe variant of the disease. It can involve any organ system of the body. Although central nervous system and vascular involvement tend to occur less frequently, they are the commonest cause of mortality. We present a case of a 30-year-old man referred with suspicion of cerebral venous sinus thrombosis to our hospital and subsequently diagnosed with BD. The patient developed, despite being on immunosuppression and anticoagulation, extensive arteriovenous thrombi of lower limbs requiring catheter-directed thrombolysis with continuous 24-hour infusion of tissue plasminogen activator for refractory right lower limb venous thrombosis and placement of inferior vena cava filter to prevent pulmonary embolism. Later disease remission was achieved with rituximab.

## Introduction

Behcet’s disease (BD) was first described by Hulusi Behcet in 1937 [1]. It is a chronic, relapsing multisystem inflammatory disorder with a worldwide distribution and mostly affects people in third and fourth decades of life. It is common in Far East, Mediterranean region and the Middle East [2,3]. Although both genders are equally affected, men tend to have more severe disease. The pathogenesis of the disease is a combination of autoimmunity and autoinflammation. The main pathology being systemic perivasculitis with neutrophilic infiltration and swelling of endothelium. Vascular involvement occurs in 8%-13% of cases of BD [4]. Of this, the most common is venous involvement in which lower limb venous thrombosis is the most prominent presentation. Arterial involvement is rare with arteriovenous involvement being rarer [5]. Mortality and long-term morbidity of BD are mainly due to central nervous system (CNS) and vascular involvement [6]. We present a case of BD with multiple arteriovenous thrombosis and refractory thrombosis of right lower limb requiring catheter-directed thrombolysis using 24-hour continuous tissue plasminogen activator (tPA) infusion.

## Case presentation

A previously healthy, 30-year-old man was referred from a small hospital to state-of-the-art tertiary care, Military hospital Rawalpindi, with the suspicion of cerebral venous sinus thrombosis (CVST) on the basis of MRI report without accompanying MRI films. He initially presented with throbbing headache, vomiting and bilateral blurring of vision for five days. Any preceding history of fever, diarrhea or upper respiratory tract infection was not present. Past medical history revealed recurrent painful oral and genital ulcers for the last three years. Family history did not reveal any inflammatory or vasculitic disorders.

On examination, he was afebrile with regular pulse of 78 beats/minute, blood pressure 125/85 mmHg, respiratory rate 21 breaths/minute and Glasgow Coma Scale (GCS) 15/15. His higher cognitive functions were normal. Visual acuity was 6/12 for both eyes. Grade 2 papilledema was observed on fundoscopy. Moreover, scrotal scars were also noticed in genital area. Rest of the systemic examination, including CNS and cranial nerves, remained unremarkable.

CT scan brain done urgently at our facility at time of admission was unremarkable. Due to the suspicion of CVST based on previous MRI report and history of headache and blurred vision, immediately CT venography (CTV) brain was carried out. CTV brain confirmed the diagnosis of CVST, mainly involving bilateral transverse sinuses.

He was immediately started on therapeutic dose of subcutaneous enoxaparin twice daily and later on switched to warfarin. Testing for factor V Leiden, proteins C and S, antithrombin III and prothrombin gene mutations as part of thrombophilia screen was carried out before starting anticoagulation and later came out negative. Angiotensin-converting enzyme, autoimmune screen including extractable nuclear antibodies, antiphospholipid antibodies, anticardiolipin antibodies, antinuclear antibodies and anti-dsDNA antibodies were unremarkable. Syphilis, Brucella, hepatitis B virus and hepatitis C virus serology were normal. Flow cytometric assessment did not reveal any deficiency of cluster of differentiation (CD) 55 and CD 59. Serum complement levels were normal. Complete blood picture, renal and liver function tests were also unremarkable. Cerebrospinal fluid (CSF) analysis was unremarkable; however, opening pressure was 250 mmHg. Serum homocysteine levels were slightly raised 27.9 µmol/l (normal <15 µmol/l). D-dimers were 350 ng/ml (normal < 250 ng/ml). Dermatology review revealed positive pathergy test. Considering the International Criteria for Behcet’s Disease (ICBD), in our patient the diagnosis of BD was made and CVST was considered due to BD.

**Figure 1:**
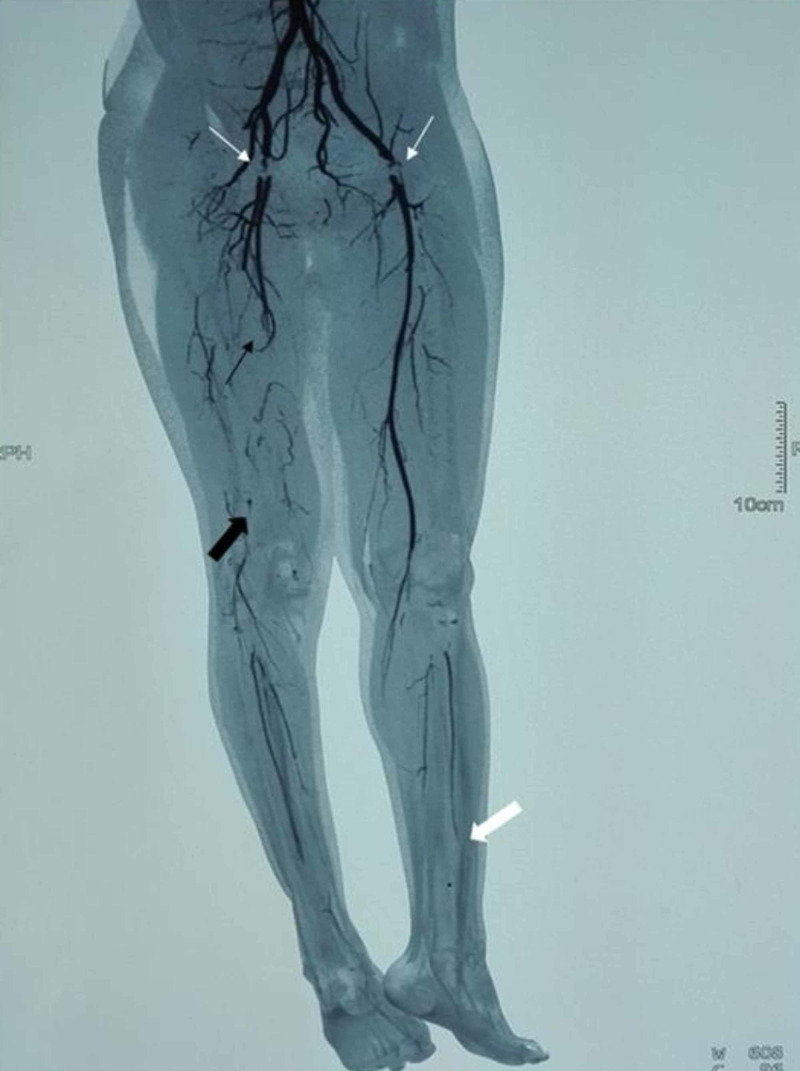
CT arteriography of lower limb reconstructed image Filling defects are visible throughout the length of common femoral arteries bilaterally (thin white arrows). A filling defect is evident in distal half of right superficial femoral artery (thin black arrow) and whole length of right popliteal artery (thick black arrow). Non-opacification of proximal part of left profunda femoris and left superficial femoral artery, and normal flow in the distal parts of these arteries is evident. In the left limb, anterior tibial artery is attenuated distally (thick white arrow) and dorsalis pedis is absent.

The patient underwent rapid immunosuppression with five sessions of plasmapheresis and intravenous methylprednisolone body weight once daily for five days along with already ongoing anticoagulation. Subsequently, the patient was treated with intravenous cyclophosphamide every three weeks along with intermittent steroids regime. While the patient was on this regime initially, he improved, headaches settled and vision improved to 6/6 bilaterally with resolution of papilledema. He remained asymptomatic for initial four months, when he developed claudication of the right lower leg. Doppler ultrasonography (USG) and CT arteriography of lower limbs and mesenteric vessels confirmed thrombosis in common femoral arteries bilaterally, popliteal artery of right lower limb and distal arteries of left lower limb with partial collateral supply already established (Figure 1). Vascular consultation was carried out in light of which the patient was managed conservatively as collaterals had already developed for arterial channels.

Despite being on anticoagulation with warfarin and international normalized ratio (INR) in therapeutic range throughout the course of disease along with immunosuppression with cyclophosphamide, thrombosis extended up to right external iliac artery. At the same time, he also developed deep vein thrombosis (DVT) in right lower limb extending to external iliac vein confirmed on Doppler USG of lower limbs. This resulted in severe right limb and scrotal edema along with cutaneous ulcers on right lower leg with excruciating pain.

**Figure 2:**
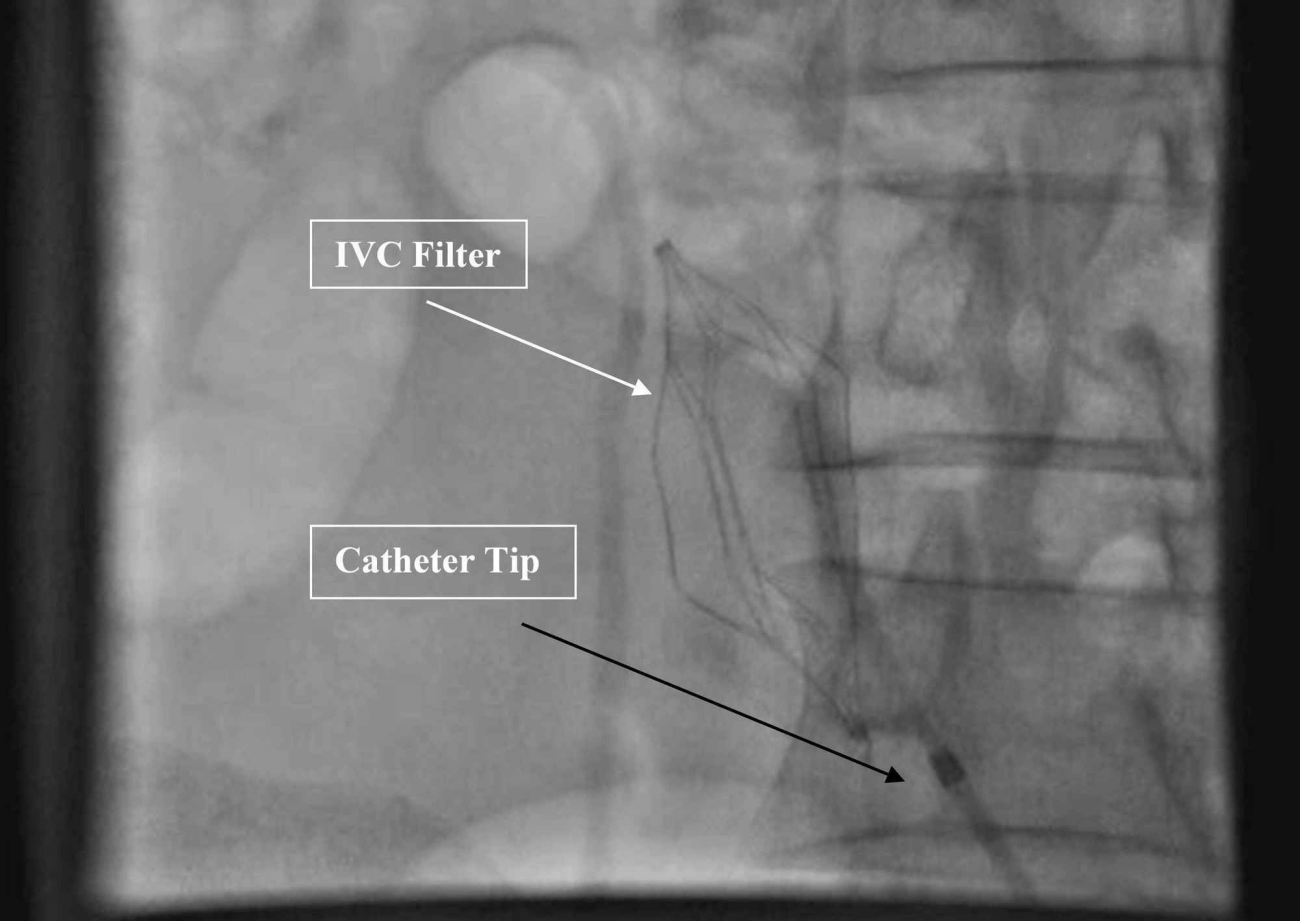
Placement of IVC filter through left femoral vein approach IVC filter is placed through left femoral vein approach to prevent pulmonary embolism. The white arrow indicates IVC filter, while the black arrow points at the tip of catheter. IVC, inferior vena cava

At this time, multidisciplinary approach, involving vascular surgeon, interventional radiologist, neurologist and rheumatologist, was carried out. It was concluded that subcutaneous leg edema was due to venous occlusion and congestion. The arterial system had developed collaterals, so it was decided to open venous channels first, as removing arterial thrombus could aggravate the subcutaneous edema by increasing flow. Keeping in view the patient’s extensive thrombosis, first inferior vena cava (IVC) filter was placed (Figure 2) through left femoral vein approach to prevent pulmonary embolism.

**Figure 3:**
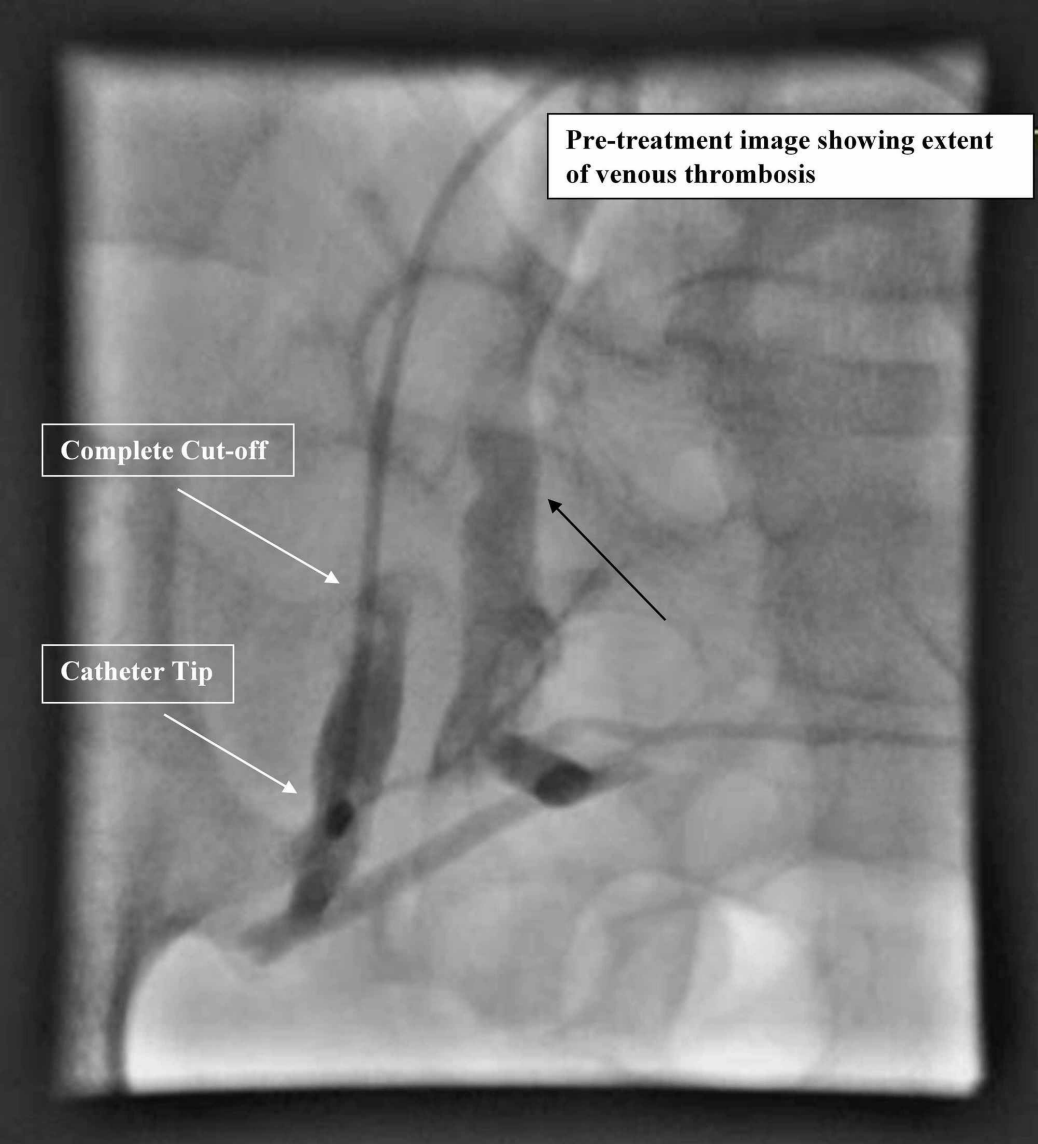
Pre-procedure image with complete cut-off in right external iliac vein Pre-procedure image with complete cut-off of contrast flow in right external iliac vein on post-contrast image (upper white arrow). Blood is reaching IVC via alternate pathway through right internal iliac vein (black arrow). Tip of catheter is also visible (lower white arrow). IVC, inferior vena cava

Next, a catheter with laterally placed holes was inserted into the thrombus in right external iliac vein and right common femoral vein via left femoral vein approach. There was complete cut-off of flow due to thrombosis in right external iliac vein on pre-treatment angiogram (Figure 3). 

**Figure 4:**
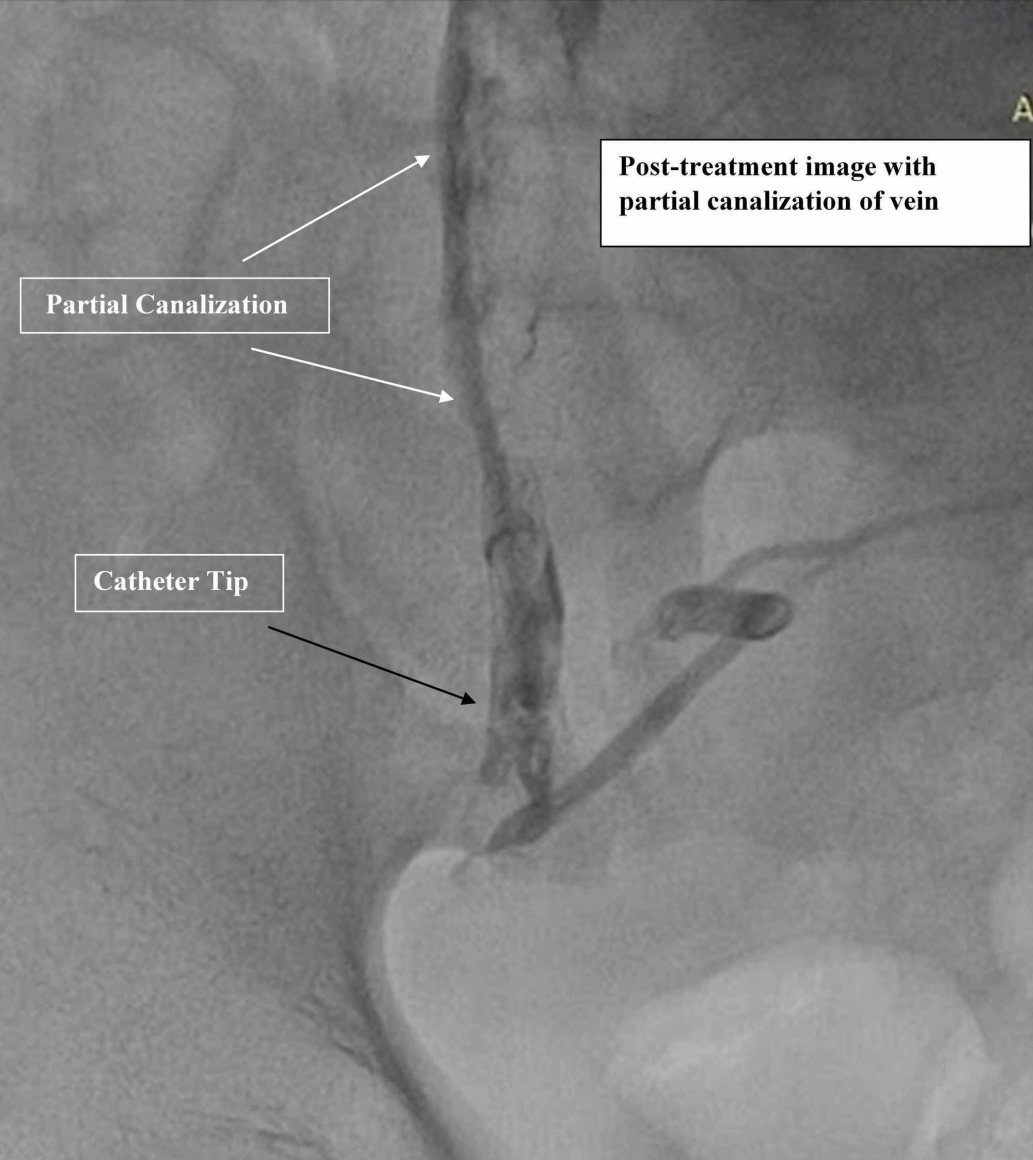
Post-procedure image with partial recanalization of right external iliac vein After 24 hours of tPA infusion through catheter, repeat angiography shows partial recanalization of right external iliac vein (white arrows) of right lower limb on post-contrast image. tPA, tissue plasminogen activator

Infusion of tPA 1 mg/h for 24 hours was continued locally into the veins of right leg through the catheter. After 24 hours repeat catheter-based angiography showed partial recanalization of proximal veins of right lower limb (Figure 4) with resultant complete resolution of scrotal and leg edema in few days. Despite complete resolution of edema, ulcers persisted in the right lower leg with severe pain. 

The patient underwent another session of plasmapheresis for five days to achieve remission. However, at this stage leg ulcers got infected despite being on broad-spectrum antibiotics and finally right above knee amputation was done to eliminate source of sepsis and to settle devastating pain in right lower leg. Four weeks later, after the sepsis settled and wound partially healed, the patient was injected with two doses of one-gram rituximab two weeks apart, considering the refractory nature of BD despite on steroids and cyclophosphamide (cumulative dose nine grams given over six months). The patient developed remission after rituximab and was discharged after two months with walking aid. Cyclophosphamide and steroid were stopped after initiation of rituximab. The patient remained on warfarin and immunosuppression with rituximab six monthly doses and underwent regular follow-up to monitor disease activity. At one year, he was ambulant with support, with IVC filter still in place. After one and a half years, the patient did not show up for routine visit and was lost to follow-up.

## Discussion

BD can have a wide variety of manifestations. The major being recurrent oral aphthae, genital ulcerations, ocular pathology and skin manifestations. Neurological and vascular involvement are comparatively rare, albeit they are the major cause of long-term morbidity and mortality in BD [6].

Neuro-Behcet’s disease (NBD) can involve both the CNS and peripheral nervous system. CNS disease has two spectrums: parenchymal and non-parenchymal involvement. In parenchymal NBD, brainstem is predominantly involved along with which spinal cord and/or cerebral hemispheres may be involved [7]. Patients present with pyramidal signs, hemiparesis, behavioral changes, sphincter dysfunction and/or impotence. Non-parenchymal disease occurs secondary to vascular involvement. These patients present mainly with headache as a result of intracranial hypertension having papilledema and unilateral/bilateral VI nerve palsy [7]. Our patient presented with headache, vomiting and bilateral blurring of vision, which was subsequently found out to be due to CVST. In a cohort study, 8% of BD patients were reported to have CVST [8].

Vascular involvement in BD presents a dynamic challenge to physicians as it can present as thrombosis, aneurysm and dissection of arteries or with thrombosis of veins. The cause of thrombosis is due to inflammation within the vessel wall rather than a hypercoagulable state [9]. Hence, immunosuppression is essential to prevent relapse of venous thrombosis in BD [10]. Despite being on immunosuppression and anticoagulation, patients can still develop arterial and venous thrombi as exhibited by our case. As such, routine follow-up of patients along with patient education is essential to quickly detect any further progression of disease to reduce morbidity and mortality.

BD runs a relapsing and remitting course. The general principle of management is to treat the manifestations as soon as they arise and to attenuate excessive inflammatory response with use of immunosuppressive therapy, such as adalimumab, cyclophosphamide, azathioprine, methotrexate and cyclosporine [11]. For refractory limb venous thrombosis in BD, catheter-directed thrombolysis can be a consideration as employed in our case.

## Conclusions

Although BD is well known, but when it initially presents with thrombosis, it becomes difficult to make a definitive diagnosis. Detailed history taking is essential for coming at a correct diagnosis. Aggressive immunosuppression along with anticoagulation is the main stay of treatment for thrombosis in BD. Even with adequate treatment thrombosis can recur or progress, hence the need for close patient follow-up. Our case sheds light on the role of catheter-directed thrombolysis for the treatment of refractory thrombosis in BD.

## References

[1] Behcet H (1937). Uber rezidivierende, aphthose, durchein virus verusachte gaschwure am mund, am auge und an den genitalien. Dermat Wochsch.

[2] Yurdakul S, Hamuryudan V, Yazici H (2004). Behçet syndrome. Curr Opin Rheumatol.

[3] Mahr A, Maldini C (2014). Epidemiology of Behçet's disease. Rev Med Interne.

[4] Davatchi F, Shahram F, Chams-Davatchi C (2010). Behcet’s disease: from east to west. Clin Rheumatol.

[5] Kabbaj N, Benjelloun G, Gueddari FZ, Dafiri R, Imani F (1993). Vascular involvements in Behçet disease. Based on 40 patient records (Article in French). J Radiol.

[6] Kural-Seyahi E, Fresko I, Seyahi N (2003). The long-term mortality and morbidity of Behcet syndrome: a 2-decade outcome survey of 387 patients followed at a dedicated center. Medicine (Baltimore).

[7] Akman-Demir G, Serdaroglu P, Tasçi B (1999). Clinical patterns of neurological involvement in Behçet's disease: evaluation of 200 patients. Brain.

[8] Saadoun D, Wechsler B, Resche-Rigon M (2009). Cerebral venous thrombosis in Behcet's disease. Arthritis Care Res.

[9] Calamia KT, Schirmer M, Melikoglu M (2005). Major vessel involvement in Behçet disease. Curr Opin Rheumatol.

[10] Desbois AC, Wechsler B, Resche-Rigon M (2012). Immunosuppressants reduce venous thrombosis relapse in Behcet's disease. Arthritis Rheumatol.

[11] Emmi G, Vitale A, Silvestri E (2018). Adalimumab-based treatment versus disease-modifying antirheumatic drugs for venous thrombosis in Behçet's syndrome. Arthritis Rheumatol.

